# Predicting sudden cardiac death in heart failure with reduced ejection fraction

**DOI:** 10.1007/s10741-026-10619-1

**Published:** 2026-03-30

**Authors:** Norayr Mkrtchyan, Alirameen Akram, Neal Dixit, Alexander Hajduczok, Antoinette Birs

**Affiliations:** 1 UC Davis School of Medicine, Sacramento, CA USA; 2 Division of Cardiovascular Medicine, UC Davis, Sacramento, CA USA; 3 Department of Medicine, UC San Diego, San Diego, CA USA; 4 Division of Cardiovascular Medicine, UC San Diego, San Diego, CA USA

**Keywords:** Heart failure, Sudden cardiac death, Risk stratification, Arrythmias, Cardiac imaging, Biomarkers

## Abstract

Sudden cardiac death (SCD) accounts for up to half of all mortality in patients with heart failure with reduced ejection fraction, yet contemporary clinical practice continues to rely primarily on left ventricular ejection fraction (LVEF) despite its well-recognized limitations as a predictor of arrhythmic risk. Many patients who experience SCD have only mildly reduced or even preserved LVEF, while a substantial proportion with severely reduced LVEF never develop life-threatening arrhythmias, underscoring the need for more precise risk assessment tools. This review synthesizes current evidence on clinical features, comorbidities, biomarkers, electrocardiographic abnormalities, and advanced imaging markers that independently inform SCD risk, including high-risk indicators such as syncope, chronic kidney disease, ventricular arrhythmias, impaired global longitudinal strain, and myocardial fibrosis on cardiac MRI. We also summarize emerging multiparametric risk models that integrate these variables into clinically usable frameworks, offering more individualized risk estimates than ejection fraction alone. Finally, we outline contemporary prevention strategies that optimize guideline-directed medical therapy and targeted device therapy, while discuss the evolving role of precision-medicine approaches in guiding ICD decision-making. Collectively, these data highlight the importance of moving beyond LVEF-centric algorithms toward more nuanced, multimodal strategies to better identify high-risk patients and reduce the ongoing burden of SCD in heart failure patients.

**Supplementary Information:**

The online version contains supplementary material available at 10.1007/s10741-026-10619-1.

## Introduction

Among patients with heart failure with reduced ejection fraction (HFrEF), it is estimated that approximately 30–50% of all deaths can be attributed to sudden cardiac death (SCD) [1–4]. Left ventricular ejection fraction (LVEF) is currently the main clinical criterion used in the decision to place a primary prevention implantable cardioverter-defibrillator (ICD) despite the abundance of evidence that LVEF can either underestimate or overestimate risk of SCD [5]. There is a growing acknowledgement of the need for precision medicine in this complex and heterogeneous disease state in which chronicity, mechanism of insult, and comorbid factors modify individual risk beyond that of ejection fraction. A better understanding of these factors can inform clinicians’ decisions that may reduce risk of SCD, including optimization of guideline-directed medical therapy (GDMT), coronary revascularization, or proceeding with advanced therapies for end-stage cardiomyopathy, such as left ventricular assist device (LVAD) placement and cardiac transplantation. While several risk scores have been developed to identify HFrEF patients at risk of SCD, they are often underused, can be cumbersome to use in clinical practice, and require a variety of clinical variables that may not be available during a patient encounter [6–8]. 

**Fig. 1:**
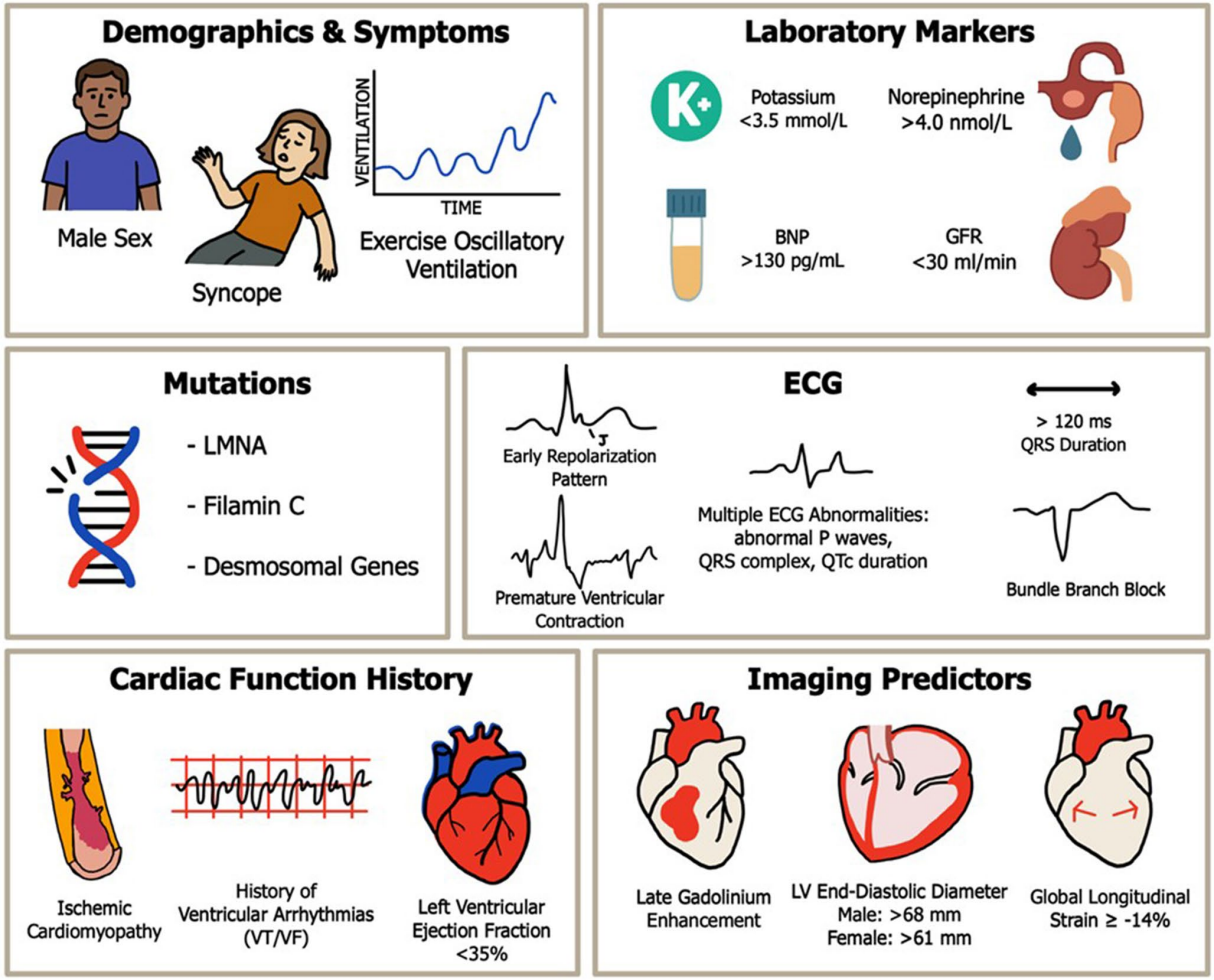
High-yield risk stratifiers

The purpose of this review is to synthesize key predictors of SCD in HFrEF, evaluate available risk stratification tools, and provide clinicians with practical guidance for individualizing patient care (Fig. 1).

## Methods

A review of literature on SCD in HFrEF was conducted from January 1, 1975, to August 1, 2025, using a MEDLINE (PubMed) database search. The search strategy used the terms (“Heart Failure”[Mesh] OR “heart failure” OR “HFrEF” OR “left ventricular dysfunction”) AND (“Sudden Cardiac Death”[Mesh] OR “sudden cardiac death” OR “sudden death” OR “arrhythmic death”). Cohort studies, randomized controlled trials, case-control studies, and systematic reviews examining risk factors, predictors, and prevention strategies for SCD in HFrEF were identified and compiled. Details of the full search strategy, inclusion and exclusion criteria, and search results are provided in Supplementary File 1. Select studies with a high level of evidence were highlighted in Table 1.

**Table 1:** Clinical factors associated with risk of sudden cardiac death in HFrEF

Risk Factor	High-Risk Criteria	Magnitude Increase in Risk of SCD
*Symptoms*		
Syncope	Any history of syncope	3.8X [9]
Abnormal CPET	VE/VCO_2_ *≥* 37.0	5.4X [10]
	Exercise oscillatory Ventilation (EOV)	< 1% absolute risk of SCD if no EOV [10]
*Cardiac Function History*		
Etiology of HF	Ischemic vs. non-ischemic	3.0X [11]
Left Ventricular Ejection Fraction	LVEF < 35% compared to HF with LVEF > 35%	1.7X [12] at 5-year mark
Ventricular Arrhythmias	Previous VT/VF episode	2-3X [13]
	Non-sustained VT (risk increases with more frequent episodes, shorter cycle length, and coexistence with complex and frequent PVCs)	> 2.8X [14]
	Inducibility on electrophysiology study	1.5X [15]
*Laboratory Markers*		
BNP (NT-proBNP)	> 130 pg/mL (equivalent NT-proBNP ~ 780 pg/mL)	19X [16]
Norepinephrine Levels	> 4.0 nmol/L	2.9X [17]
Potassium Levels	< 3.5 mmol/L	4.0X [18]
eGFR	< 30 ml/min/1.73 m^2^	1.7X [19]
LMNA or Desmosomal Genes	Presence of mutation	3.0X [20]
*ECG Abnormalities*		
Heart Rate Turbulence	Abnormal turbulence onset and turbulence slope	2.3X [21]
Early Repolarization Pattern	J-point elevation ≥ 0.1 mV in at least 2 inferior or lateral leads	4.5-7.2X [22]
Left Bundle Branch Block	Presence of a bundle branch block elevates risk	1.4X [23]
Multiple ECG Abnormalities	≥ 2 of: abnormal P waves, QRS prolongation, QTc duration, T-peak-to-T-end interval, PR interval	3.7X [24]
ECG Abnormalities in Non-Ischemic Cardiomyopathy	J wave in the inferior leads	4.0X [25]
ECG Abnormalities in Ischemic Cardiomyopathy	Fragmented QRS in inferior lead	2.7X [25]
*Imaging Predictors*		
Late Gadolinium Enhancement	Presence vs. Absence	3.9X [26]
	Late Gadolinium Enhancement + QRS duration *≥* 120 ms	9.5X [27]
Left Ventricular End-Diastolic Diameter	> 68 mm in men, > 61 mm in women	2.7X [28]
Global Longitudinal Strain	*≥* -14%	1.9X [29]
Mechanical Dispersion	*≥* 75ms	9.9X [29]
*Risk Scores*		
ADMIRE-HF Score	> 15 associated with high SCD risk	4.3X [6]
PARADIGM-HF Model	Upper vs. Lower quartile	7.7X [30]
ATMOSPHERE Model	NT-proBNP (≥ 1204 pg/mL), QRS duration (≥ 120 ms), and history of MI predicts risk	2.5-4.2X [7]

*Abbreviations*: *BNP* B-type natriuretic peptide, *CPET* Cardiopulmonary Exercise Test, *ECG* Electrocardiogram, *EOV* Exercise Oscillatory Ventilation, *HF* Heart Failure, *LVEF* Left Ventricular Ejection Fraction;, *NT-proBNP* N-terminal pro-B-type natriuretic peptide, *PVC* Premature Ventricular Contractions, *SCD* Sudden Cardiac Death, *VCO₂* Carbon Dioxide Production, *VE* Minute ventilation, *VF* Ventricular Fibrillation, *VT* Ventricular Tachycardia

## Discussion

There is controversy as to what is considered a SCD in (heart failure) HF since many HF patients have significant preexisting symptoms and a high anticipated short-term mortality rate. As Milton Packer describes, “the definition of SCD has now evolved to mean any death in an HF patient who was anticipated to have at least several months to years of survival.” [1] The mechanism of SCD in HFrEF patients is most often tachyarrhythmia or acute mechanical failure, usually preceded by pulseless electrical activity or bradyarrhythmia, with the former being more common in less advanced HFrEF patients [1, 31]. The proximal trigger for SCD is often impossible to identify, which explains why so many of the deaths in HFrEF patients are unexpected. However, identification of risk factors in symptomatology, comorbidities, and objective laboratory, imaging, and electrocardiographic findings can improve a clinician’s gestalt of a HFrEF patient’s risk of SCD.

In this review, we first discuss patient symptomatology and comorbidities that have been associated with an increased risk of SCD. These include both modifiable and non-modifiable characteristics. We then examine objective biomarkers, ECG and imaging findings associated with SCD. Additionally, we review validated risk scores that have integrated multiple variables to stratify the risk of SCD. Lastly, we discuss current risk strategies for SCD prevention and treatment.

## Symptoms

Patient symptoms provide important clinical signals to identify patients at high risk for SCD. Among these, syncope and exertional intolerance offer significant prognostic insights.

### Syncope

Syncope is the symptom most strongly associated with mortality in HFrEF patients. In a cohort study of 491 patients with severe HF, the rate of SCD was higher in those with a history of syncope (45%) compared to those without (12%) within a year of follow-up [9]. This association was irrespective of etiology of syncope and remained an independent predictor of SCD in multivariate analysis. Syncope in HFrEF may be due to arrhythmias (e.g., ventricular tachycardia or fibrillation), bradycardia, or hemodynamic instability resulting from impaired cardiac output or autonomic dysfunction. Given this strong link with poor outcomes, routinely evaluating for a history of syncope is a useful clinical tool for predicting SCD in HF patients [32, 33]. 

### Exertional intolerance

Classification of dyspnea in HF patients has traditionally been performed by the New York Heart Association (NYHA) classification. However, a direct correlation between increasing NYHA class and risk of SCD does not exist [34, 35]. In one study of patients with dilated cardiomyopathy (DCM) and an ejection fraction (EF) ≤ 35%, NYHA class I patients had similar risks of SCD or ventricular arrhythmias (VA) as those in NYHA classes II or III. Cardiopulmonary exercise testing (CPET) provides an objective, reproducible method for evaluating HF symptoms and stratifying SCD risk. The most important predictor of SCD in CPET is exercise oscillatory ventilation (EOV), which is an abnormal, irregular breathing pattern during exercise [10]. HFrEF patients without EOV had a very low risk of SCD over a period of several years compared to patients with an abnormal pattern who had a nearly 20% yearly risk of SCD. Risk of SCD persisted even after adjustment for peak VO2 and VE/VCO_2_ slope, both of which correlate with overall prognosis [10]. 

## Comorbidities

Comorbidities significantly modify SCD risk in HFrEF patients. Chronic kidney disease (CKD), ventricular arrhythmias, and ischemic cardiomyopathy notably heighten this risk, independent of traditional metrics such as LVEF.

### Chronic kidney disease

CKD complicates heart failure management and can elevate SCD risk. Among patients with HF, reduced kidney function, as measured by estimated glomerular filtration rate (eGFR), is an independent predictor of SCD. Specifically, patients with an eGFR < 30 ml/min/1.73 m² had a hazard ratio (HR) of 1.73 (95% CI: 1.11–2.70, *p* = 0.01) for SCD compared to those with better kidney function [19]. This predictive value is enhanced when eGFR is combined with LVEF. For example, patients with both eGFR < 30 ml/min/1.73 m² and LVEF ≤ 35% had a significantly higher risk of SCD (HR: 5.17; 95% CI: 3.01–8.86, *p* < 0.01).

### Ventricular arrhythmias

Ventricular arrhythmias such as ventricular fibrillation (VF), sustained ventricular tachycardia (VT), non-sustained VT (NSVT), and premature ventricular contractions (PVCs) are major causes of SCD in patients with left ventricular (LV) dysfunction. The risk of SCD is especially high in patients who have sustained VT or VF, frequent NSVT, or a positive electrophysiologic study.

The risk of recurrent VT/VF after a first episode is substantial; data from the MADIT I and II trials show a 46% recurrence rate within 1 year and 60% within 3 years among HF patients with a primary prevention ICD [13, 36]. In the AVID trial (1997), the incidence of arrhythmic death in patients with a history of VT/VF was 8% in patients in the non-ICD arm at 1 year [14]. Meanwhile, the risk of SCD in HFrEF patients without a history of VT/VF was less than 3% based on data from SCD-HeFT [37]. 

NSVT is also strongly associated with risk of SCD. The presence of NSVT on a 24-hour Holter monitor portended a 2.77-fold increase in the risk of SCD in a pre-ICD prospective cohort study compared to those without [38]. This was not modified by the etiology of HF. Risk increases as NSVT episodes increase and cycle length of the tachycardia decreases [39]. 

Inducibility on electrophysiology study also portends an elevated risk of SCD. In the MUSTT trial, post-MI patients with an LVEF *≤* 40%, NSVT, and inducibility on electrophysiology study had a 1.5 times higher risk of SCD at 5 years than patients with no inducibility [15]. 

PVC morphology and burden is known to predict SCD risk in HFrEF patients [40, 41]. In post-MI patients, PVCs that demonstrated complex characteristics which included “R-on-T” PVCs, PVC couplet, multiform PVCs, and bigeminy pattern PVCs, increased the risk of SCD by > 3 times compared to those with non-complex or no PVCs [42]. Another study in a similar population showed that PVC burden > 10 per hour was an independent predictor of SCD although risk increased with more complex PVCs [43]. However, whether PVCs have an independent predictive value beyond their association with NSVT is less clear, especially in non-post-MI patients [44]. 

### Ischemic vs. non-ischemic cardiomyopathy

An analysis of five landmark ICD trials showed that patients with ischemic cardiomyopathy (ICM) vs. non-ischemic cardiomyopathy (NICM) had similar rates of VT/VF and appropriate ICD discharge rate [11]. However, patients with ICM had a 1.87 times increased risk of mortality in adjusted multivariable analysis. Rates of sudden death were also three times higher in unadjusted analyses in the ICM compared to the NICM.

### Right ventricular dysfunction

Data from over 5,000 patients from The Mayo Clinic cardiac care unit database found a significant prevalence of right ventricular systolic dysfunction with 15% having mild and 17% having moderate-severe dysfunction [45]. Both sub-cohorts were found to have a higher risk of sudden death with a HR of 1.57 and 1.91, respectively, even after accounting for baseline comorbidities and LVEF. Notably, moderate-severe right-ventricular dysfunction has remained a strong predictor of sudden death, even in patients with a preserved LVEF, and those already with ICDs, underscoring its prognostic value.

## Non-modifiable characteristics

Some of the strongest indicators of prognosis for SCD lie in factors that are inherent to the individual patient and non-modifiable.

### Race and sex

In analyses of recent clinical trials, Black patients have consistently had higher rates of SCD compared to White patients, but confounding socioeconomic factors require further investigation [28, 46, 47]. In an analysis of PARADIGM-HF, Asian patients had higher rates of SCD but the significance of this is uncertain and may be due to chance due to low numbers [47]. Female sex has been generally mildly protective against SCD [48, 49]. 

### Genetic predisposition

Patients with dilated cardiomyopathy and mutations in LMNA, filamin C, or desmosomal genes are highly predisposed to ventricular arrhythmia and SCD [50]. The risk of SCD or VA was around 3 times higher in DCM patients with LMNA or desmosomal gene mutations compared to DCM patients without an identifiable mutation or gene mutation not associated with additional risk of SCD [20, 50]. Because of this, increased risk guidelines have incorporated higher LVEF cutoffs for primary prevention ICD implantation, up to 45%, for DCM patients with high-risk mutations [50]. Identification of high-risk mutations should prompt more aggressive monitoring and possibly earlier consideration of ICD placement.

## Biomarkers

Circulating plasma biomarkers provide objective, quantitative tools for assessing SCD risk. Circulating catecholamines, NT-proBNP, troponin, and potassium level are particularly valuable.

### Circulating catecholamines

​​Persistently elevated norepinephrine (NE) levels are a strong predictor of SCD in patients with HFrEF. In HFrEF patients, NE levels above 4.0 nmol/L were associated with a nearly threefold increased risk of cardiogenic death, including SCD (HR: 2.91; 95% CI: 1.08–7.33; *P* = 0.015) [17]. Similarly, epinephrine levels and intra-lymphocyte cAMP content were independently linked to increased mortality risks, suggesting a critical role of heightened sympathetic activity.

### Natriuretic peptides

Natriuretic peptides such as NT-proBNP and BNP have emerged as strong, independent predictors of SCD, regardless of LVEF [46, 51–54]. A study evaluating over 450 patients with LVEF ≤ 35% found that BNP levels independently predicted SCD risk. Notably, patients with BNP levels exceeding 130 pg/mL (equivalent to NT-proBNP of ~ 780 pg/mL) face an elevated risk of SCD, with an 81% sudden death-free survival rate over three years, compared to 99% in those below this threshold [16]. In a post-MI population, a BNP level of ~ 80 pg/mL (equivalent to NT-proBNP of ~ 480 pg/mL) combined with an LVEF < 40% identified 85% of patients who suffered SCD. However, the DANISH trial suggested that those with a lower than the median NT-proBNP (1,177 pg/mL), had similar rates of SCD compared to those above the median, but derived greater benefit from ICD therapy. However, this is in conflict with data from PARADIGM-HF and other studies, which suggest higher natriuretic peptide levels associated with SCD and tachyarrhythmia therapy [46]. Overall, the general direction of the data favors a positive correlation between natriuretic peptides and SCD, but whether ICD therapy attenuates this risk is unclear.

### Troponin

Troponin, specifically high-sensitivity cardiac troponin (hs-Tn), has been established as a significant biomarker for risk assessment in heart failure patients [55, 56]. In a study of 744 patients followed for 5 years, hs-Tn was associated with SCD and patients experiencing SCD had a baseline hs-Tn of 31.6 ng/L compared to patients alive at the end of 5-year follow up (14.7 ng/L) [57]. The risk of SCD increased by 65% per 1 standard deviation of the log-transformed hs-Tn level. Elevated hs-Tn levels in HFrEF patients reflect ongoing myocardial damage, serving as a predictor of adverse outcomes, including SCD. However, more data is needed to understand the thresholds at which risk increases.

### Potassium

Potassium levels have shown a U-shaped association with death in heart failure patients with both low and high levels increasing risk [18]. Potassium levels below 3.5 mmol/L have been associated with disproportionate risk of ventricular arrhythmias and SCD due to prolonged action potentials and increased excitability. Correction of hypokalemia to a target range of 4.0–5.0 mmol/L has been associated with improved outcomes, as evidenced by the reductions in event rates in trials like EMPHASIS-HF and RALES [58]. Additionally, potassium correction to target the high-normal range (4.5-5.0 mmol/L) in ICD carriers at high-risk for arrhythmias reduced the composite risk of arrhythmias, hospitalization, and all-cause mortality compared to standard care in the POTCAST trial [59]. 

## Electrocardiographic changes associated with SCD

ECG abnormalities such as heart rate turbulence, early repolarization, prolonged QRS, and fragmented QRS offer simple yet powerful tools to identify patients at increased SCD risk [60]. 

### Heart rate turbulence

Heart rate turbulence refers to a characteristic short-term fluctuation in sinus cycle length after a PVC. In a healthy heart, it consists of an initial acceleration of the heart rate after a PVC, followed by gradual deceleration to baseline. Turbulence onset describes the percentage change of the first two RR intervals after the PVC compared to the last two sinus RR intervals that preceded it (negative slopes correlating with healthier hearts). Turbulence slope describes the steepest positive slope of a regression line that is calculated over any 5 consecutive RR intervals among the first 20 beats after the PVC (flatter slopes correlating with sicker hearts). Abnormal turbulence is typically defined as turbulence onset greater than or equal to 0 and/or turbulence slope less than or equal to 2.5 ms/RR, suggesting impaired autonomic response after a PVC. Patients with abnormal measurements in both parameters had a 2.25 times higher rate of SCD at 3 years compared with patients with normal values in both parameters [21]. Heart rate turbulence is easily assessable with any patient with PVCs detected on ambulatory monitor, and can add a source of quantified risk stratification.

### Early repolarization patterns

Early repolarization pattern (ERP), identified as J-point elevation of ≥ 0.1 mV in at least two inferior or lateral ECG leads is associated with increased SCD risk in patients with HFrEF. In a study of 90 stable HFrEF outpatients, ERP was present in 36% of those who experienced SCD versus 6% of those who did not (*p* = 0.001). ERP independently predicted SCD risk, with a hazard ratio of 7.2 (95% CI: 2.8–18.6; *p* < 0.0001) after adjusting for age, sex, NYHA class, ischemic heart disease, and diabetes mellitus [6]. In a similar population of 132 patients, the incidence of SCD was 63% vs. 14% in those with ERP vs. those without [22]. Notably, ERP in both inferior and lateral leads conferred the highest risk. When combined with the ADMIRE-HF risk score, a validated index integrating clinical and cardiac imaging variables, ERP provides incremental prognostic value [6]. Patients with both ERP and intermediate/high ADMIRE-HF scores exhibited a 27-fold higher risk of SCD compared to those without ERP and low ADMIRE-HF scores.

#### Fragmented QRS

The presence of a fragmented QRS reflective of disrupted ventricular conduction possibly due to underlying scar was predictive of SCD in a cohort of patients with HFrEF [25]. In ischemic cardiomyopathy, a fragmented QRS in the inferior leads was associated with a 2.7 times risk of SCD.

### QRS duration

In a HF population, QRS duration of more than 120 ms was associated with 1.5 times the risk of SCD regardless of LVEF in adjusted analysis [61]. However, a secondary analysis of the EVEREST trial found similar rates of SCD between HFrEF patients with QRS duration greater or less than 120 ms [62]. A left bundle branch block was associated with SCD, increasing risk by 1.35 times in study of more than 5,000 HFrEF patients [23]. 

Another study assessing the combined prognostic value of QRS duration and late gadolinium enhancement (LGE) in patients with dilated cardiomyopathy found that these factors together significantly enhance risk stratification for SCD. Specifically, combining LGE positivity with QRS prolongation (≥ 120 ms) significantly improved risk stratification for SCD. Patients with both positive indices had a 4.29-fold higher risk of all-cause mortality (CI 1.19–15.47) and were at a 9.53-fold increased risk of SCD (CI 2.84–31.98), underscoring the prognostic value of integrating LGE with physiological markers like QRS duration. In a cohort of patients with an ICD class I indication, those with both negative LGE and a narrow QRS showed the lowest event rates for SCD, highlighting that the absence of these markers, even in patients with left ventricular ejection fraction ≤ 35%, indicates a lower risk of adverse outcomes [27]. 

#### Other ECG findings

Established markers such as QRS prolongation indicate electrical desynchrony and poor cardiac efficiency, while QT interval abnormalities and dispersion reflect repolarization heterogeneity and susceptibility to malignant arrhythmias [63]. Advanced tools like signal-averaged ECG detect low-amplitude signals indicative of myocardial scarring, while T-wave alternans and long-term Holter monitoring highlight electrical instability through beat-to-beat variability and ventricular ectopy, respectively. Building on this, Mashood et al. proposed a cumulative ECG risk score integrating six parameters: bundle branch block, abnormal P waves, QRS duration, QTc duration, Tpeak-to-Tend interval, and PR interval [24]. Their model demonstrated that patients with ≥ 2 abnormalities had a more than threefold increased risk of SCD (HR 3.74, 95% CI 1.70–8.21, *P* = 0.001), with risk proportionally increasing as more abnormalities were identified. In another study atrioventricular block was independently associated with ventricular arrhythmias and sudden death, beyond the sole predictive value that was provided with myocardial scar on CMR and LVEF [64]. 

### Imaging findings associated with SCD

Although reduced LVEF remains central to current ICD guidelines, its limitations underscore the necessity of advanced imaging markers such as speckle tracking echocardiography and cardiac magnetic resonance imaging (CMR) for refined risk stratification.

### Ejection fraction

The largest jump in the rate of mortality and SCD occurs below an LVEF of 35–40%, which formed the basis for the cutoffs in the ICD trials [65]. However, LVEF falls short as a standalone predictor. Many SCD cases occur in patients with preserved or only moderately reduced LVEF, and many individuals with severely reduced LVEF never experience life-threatening arrhythmias [66]. The Maastricht Circulatory Arrest Registry reported that 51% of SCD victims had an LVEF > 40%. Similarly, in a cohort of 2,130 myocardial infarction survivors, 67% of SCD cases occurred in individuals with LVEF > 35%. Additionally, in a study of patients who experienced SCD, upward of 30% of patients with an initial LVEF *≤* 35% experienced SCD despite a last measurement of LVEF > 35%, demonstrating that despite improvement in LVEF considerable residual risk of SCD exists [67]. The limitations extend to specificity, as reduced LVEF predicts both sudden and non-sudden mortality, complicating the identification of patients who would benefit most from ICDs. Specificity can be improved by using 3-dimensional measurements which have shown potential in predicting major arrhythmic events and have improved arrhythmic risk prediction in patients with LV dysfunction [68]. Severely increased LV end-diastolic diameter (> 68 mm in men and > 61 mm in women) is independently associated with a 2.65 times increase in SCD [28]. Overall, LVEF is an important prognostic indicator but can be non-specific and insensitive for arrhythmic events and SCD.

### Global longitudinal strain and speckle-tracking echocardiography

**Fig. 2:**
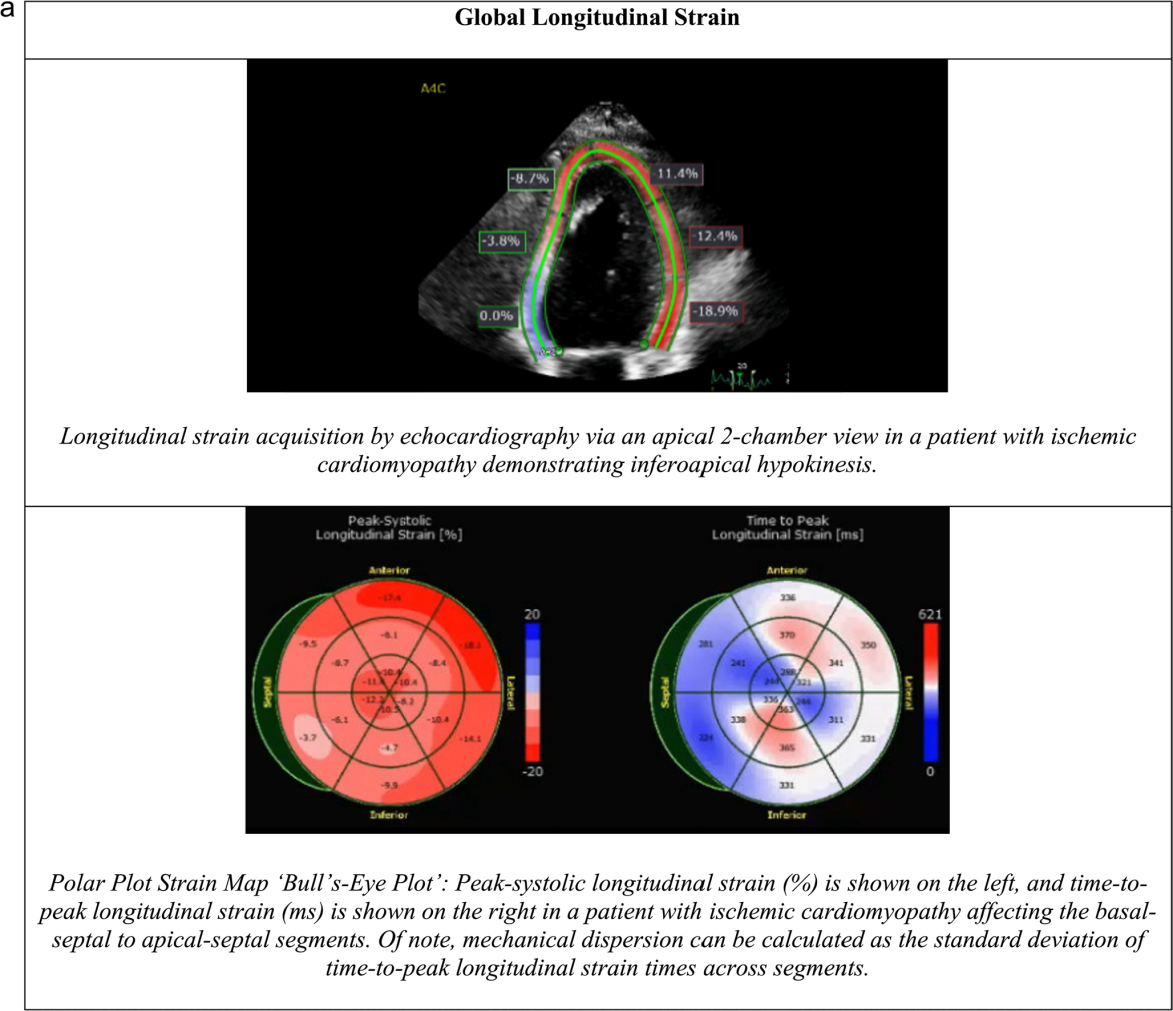

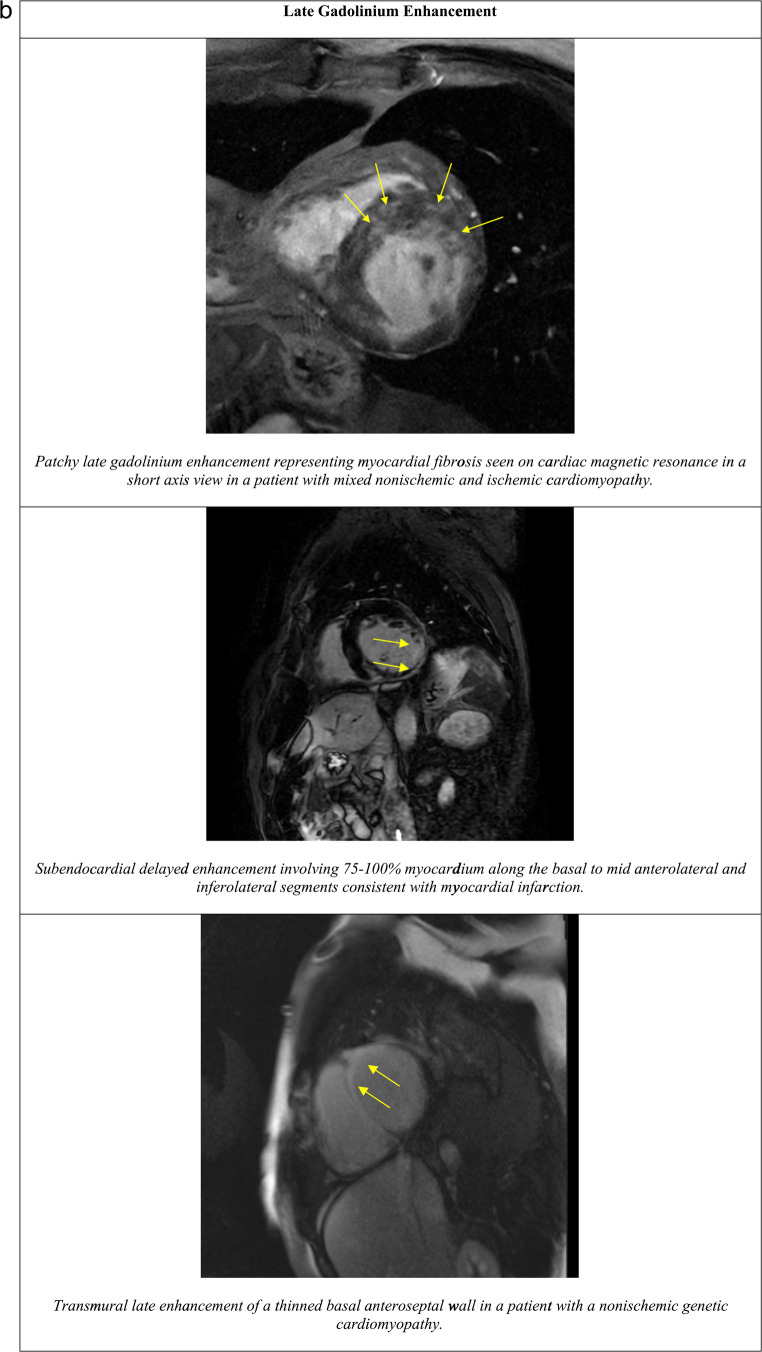
**a** Examples of global longitudinal strain in two patients with ischemic cardiomyopathy. **b** Examples of late gadolinium enhancement in various disease processes

Global longitudinal strain (GLS) is measured using 2-dimensional speckle tracking echocardiography, which quantifies myocardial deformation by tracking the motion of speckles within the heart muscle to directly measure myocardial fiber shortening and assess the degree of strain during contraction (Fig. 2). Speckle-tracking echocardiography (STE) allows for precise measurement of myocardial strain, offering a way to indirectly evaluate systolic function, especially when scarring or fibrosis is present. In a study of over 1,000 HFrEF patients, GLS outperformed conventional echocardiographic parameters, including LVEF, in predicting mortality [69].

Perry et al. followed 939 patients with HFrEF and found a GLS *≥*-14% increased the rate of SCD or ventricular arrhythmia by 1.72 times [29]. More prognostic was mechanical dispersion which measures variability in the time to maximum strain in different parts of the LV which typically represents myocardial scarring [29]. Mechanical dispersion *≥* 75ms increased risk of ventricular arrhythmia or SCD by 9.86 times.

GLS and mechanical dispersion, easily measurable via echocardiography, robustly predict mortality and arrhythmic events, potentially guiding ICD and medical therapy decisions.

### Late gadolinium enhancement

Late gadolinium enhancement on cardiac magnetic resonance imaging (CMR) identifies myocardial fibrosis by highlighting areas of delayed contrast washout, indicative of scar tissue (Fig. 2) [70–73]. 

In a systematic review by Ganesan et al. on the impact of LGE on mortality, sudden death, and major adverse cardiovascular events (MACE) in ischemic and non-ischemic cardiomyopathy, the presence of LGE was associated with a 4.25 times risk of ventricular arrhythmia and SCD which was not impacted by LVEF when dichotomized to below or above 35% or etiology of cardiomyopathy (ischemic or non-ischemic) [26, 74]. 

The extent of LGE also has an impact on risk of SCD, with increasing transmurality and affected myocardial segments portending worse prognosis [75]. However, due to significant heterogeneity in the methods for measuring and quantifying LGE, it is difficult to define thresholds for SCD risk associated with LGE. Studies have suggested that the biggest increase in risk occurs from the mere presence of LGE with additional small incremental risk accruing based on extent of LGE.

Di Marco et al. provided a simple algorithm for risk stratifying patients with non-ischemic cardiomyopathy by presence of LGE and LVEF for a composite arrhythmia endpoint [76]. The highest-risk patients with an annual event rate of 7.2%, had LGE and an LVEF ≤ 35%, while the lowest-risk patients with an annual event rate of 0.2% had no LGE and an LVEF > 20%.

Similar findings hold true for ischemic cardiomyopathy, in which even a small scar (< 2% of mean left ventricular mass) was associated with 7 times increase in MACE [77–79]. 

Overall, the data suggests that for both non-ischemic and ischemic cardiomyopathy, the presence of any LGE increases the risk of SCD regardless of EF [80]. Lower LVEF and increasing extent of LGE may provide additional secondary risk stratification.

### Ischemia on myocardial perfusion imaging

Chronic myocardial ischemia is classically viewed as a risk enhancer for arrhythmia and SCD. However, in a relatively large study of patients referred to cardiac positron emission tomography with an LVEF ≤ 35%, ischemia whether global or peri-infarct did not correlate with SCD or ICD shock. Only a severe fixed perfusion defect, likely representing transmural scar, predicted increased risk [81]. 

## Risk scores

Risk prediction models like ADMIRE-HF [6], PARADIGM-HF [7], and ATMOSPHERE [8] integrate clinical, ECG, and biomarker data into actionable scores. Clinicians can utilize these scores to personalize decisions around ICD placement and therapy intensification.

### ADMIRE-HF risk score

The ADMIRE-HF risk score integrates LVEF, cardiac I-123-metaiodobenzylguanidine (MIBG) heart-to-mediastinum ratio (H/M), and systolic blood pressure to predict serious arrhythmic events in patients with HFrEF [6]. MIBG imaging reflects cardiac sympathetic activity, with delayed H/M ratios below 1.6 indicating heightened arrhythmogenic risk [82]. Patients with high ADMIRE-HF scores (> 15) demonstrated significantly greater rates of SCD compared to those with intermediate (4–15) or low (< 4) scores (HR 4.3 for high vs. low, 95% CI 1.1–17.2, *p* = 0.009). The ADMIRE-HF score’s negative predictive value of 86% highlights its utility in identifying low-risk HFrEF patients who may not benefit from prophylactic ICD placement.

#### PARADIGM-HF and ATMOSPHERE models

Data from the PARADIGM-HF trial, externally validated in the ATMOSPHERE trial, developed a robust model for predicting SCD in HFrEF patients, incorporating NYHA class and NT-proBNP as key independent predictors [7]. The model identified additional significant factors, including male sex, Asian or Black race, prior coronary artery bypass grafting (CABG) or percutaneous coronary intervention (PCI) (protective), cancer history (protective), history of myocardial infarction, QRS duration (90–120 ms, per 5 ms increase), and left ventricular hypertrophy on ECG. Notably, NT-proBNP showed a log-linear relationship with SCD risk, highlighting its dose-dependent prognostic value. The model demonstrated moderate discrimination for SCD (Harrell’s C: 0.67) and effectively stratified patients into quartiles, with the highest risk quartile showing a markedly increased incidence of SCD at three years compared to the lowest risk quartile [30]. The exclusion of patients with ICDs improved the model’s applicability in assessing the first event risk. By integrating routinely collected clinical and ECG parameters with biomarkers, this model provides actionable insights for identifying high-risk patients who may benefit from early intervention, particularly ICD therapy.

### Notable factors not clearly associated with sudden cardiac death

Recent analyses of large, randomized trials such as PARADIGM-HF (8,000 patients [46]), ASCEND (7,000 patients [83]), and EVEREST (4,000 patients [47]), allow for a detailed look at clinical factors not clearly associated with risk of SCD. For example, age did not differ between those with SCD and those alive in PARADIGM-HF and EVEREST. Notably, the presence of an ICD was less prevalent in the patients who experienced SCD than in those who remained alive, suggesting that most patients with HFrEF die from SCD without an ICD. Additionally, prior heart failure hospitalization in PARADIGM-HF was not associated with SCD and only marginally increased risk in ASCEND and EVEREST, suggesting that many HFrEF patients may experience SCD without ever being hospitalized.

## Treatment and prevention strategies

The foundation of SCD prevention in HFrEF relies upon the optimization of GDMT and appropriate ICD placement. Additional interventions such as revascularization, antiarrhythmic therapy, and catheter ablation should be tailored to individual patient profiles and risk of SCD.

### Guideline-Directed Medical Therapy (GDMT)

All four core pillars of GDMT: beta-blockers (BB), angiotensin receptor-neprilysin inhibitors (ARNi), mineralocorticoid receptor antagonists (MRA), and sodium-glucose cotransporter 2 inhibitors (SGLT2i) are proven in clinical trials to reduce SCD risk in HFrEF patients [84, 85]. Each class is disease-modifying and promotes cardiac reverse remodeling, improving prognosis, myocardial function and decreasing risk of death.

### Implantable cardioverter-defibrillators

In HFrEF patients, ICD therapy remains a cornerstone for reducing SCD risk. While ICDs do not prevent the occurrence of VT/VF, they mitigate the risk of fatal outcomes. For patients with a history of ventricular tachycardia, ICD therapy reduces the risk of all-cause death by about 30% in meta-analysis compared to antiarrhythmic therapy alone [86]. This risk is through a reduction in arrhythmic death. For primary prevention, this benefit is also about 30% with no significant differences in treatment effect between ischemic and non-ischemic cardiomyopathy [87]. However new clinical trials in the age of modern four-pillar GDMT stand to question this benefit [88, 89]. 

### Antiarrhythmic therapy

Antiarrhythmic therapy in HFrEF primarily serves as an adjunct to ICDs for SCD prevention rather than a standalone treatment. Class IC antiarrhythmic drugs are contraindicated due to increased mortality risk. Among class III agents, dofetilide and amiodarone have a neutral effect on overall mortality but reduce arrhythmic deaths, making them suitable for suppressing ventricular arrhythmias in ICD patients [90]. 

### Catheter ablation

Catheter ablation has been shown to reduce recurrent VT episodes and ICD shocks, with trials such as VANISH [91] demonstrating a 59% reduction in VT storm and appropriate ICD shocks compared to medical escalation, however, mortality was unchanged. Combining ICD therapy with tailored procedural interventions may help manage risk in patients who face increased recurrence of arrhythmia-induced SCD.

### Revascularization

In a subgroup analysis of the STICH trial, revascularization with CABG was associated with a 27% reduction in SCD compared to medical therapy [92]. This difference was most apparent at 2 years and beyond after randomization. PCI on the other hand did not improve all-cause death or aborted sudden death compared to medical therapy in the REVIVED-BCIS2 trial [93, 94]. Revascularization with CABG in appropriate patients represents an important but less immediate method of reducing SCD in eligible HFrEF patients.

## Future directions

SCD remains a major cause of mortality in patients with HFrEF, driven by a complex interplay of structural, electrophysiological, and molecular mechanisms. Traditional risk-stratification strategies have largely centered around LVEF, yet this approach has clear limitations, as many patients who experience SCD do not meet conventional thresholds for device therapy.

Emerging biomarkers, together with novel imaging, computational tools, and artificial intelligence, offer complementary strategies to refine risk and enhance precision in SCD risk stratification.

Two ongoing trials, CMR GUIDE (NCT01918215) and BRITISH (NCT05568069), are evaluating whether myocardial scar identified by LGE can refine ICD decision-making in NICM patients with borderline (36–50%) or moderately reduced LVEF (< 35%), respectively, potentially extending preventive therapy to a broader subset of high-risk patients.

Machine-learning approaches applied to multimodal data such as biomarkers, imaging, and clinical variables may enable personalized risk prediction and integration into electronic health records for point-of-care decision support. However, these predictive models must be recalibrated to contemporary practice, as foundational datasets predate widespread implementation of ARNi and SGLT2i which may alter SCD risk trajectories.

Furthermore, expanding insights into genetic and inflammatory cardiomyopathies underscore that SCD risk in HFrEF is dynamic and etiology specific. Due to advances in precision diagnostics, there is a growing need to re-examine and re-validate risk stratification tools in the context of modern treatment paradigms.

## Limitations

A key limitation across existing studies on SCD is the inconsistent application of standardized definitions and adjudication methods. Future analyses should adopt uniform SCD definitions and standardize assessment methods to enhance data validity and comparability across studies.

While this review has focused on HFrEF, advances in medical therapy and targeted interventions have substantially reduced the residual risk of SCD, such that rates in patients with severely reduced LVEF are approaching those seen in patients with a preserved ejection fraction. Given that patients with LVEF > 40% now comprise the majority of the HF population, developing robust, novel risk-stratification strategies beyond LVEF alone is even more critical.

## Conclusion

Despite therapeutic advances, SCD remains a major challenge in HFrEF management, and reliance on LVEF alone is insufficient for accurate risk prediction. A more nuanced approach that integrates clinical characteristics, comorbidities, imaging findings, and biomarker data offers a more comprehensive means of identifying high-risk patients. Combining evidence-based strategies that reflect the multifactorial nature of SCD may enable more precise, individualized interventions and ultimately improve outcomes for this high-risk population of patients with HF.

## Supplementary Information

Supplementary Material 1.

## Data Availability

No datasets were generated or analysed during the current study.
