# Supplementary material for: Predicting sudden cardiac death in heart failure with reduced ejection fraction
Source: Heart Fail Rev. 2026 Mar 30;31(1):50. doi: 10.1007/s10741-026-10619-1 (PMC13035549; doi:10.1007/s10741-026-10619-1)
Supplement: Supplementary file 1 — Supplementary Material 1. [file 10741_2026_10619_MOESM1_ESM.docx]

*Supplementary Information*

**Search Strategy:** Sudden Cardiac Death in HFrEF: What is Known?

Procedure:

1. A manual review of the literature was initially performed to identify major studies that evaluated risk factors for sudden cardiac death in patients with heart failure.
2. A structured search strategy was then applied to Medline to identify additional relevant studies.
3. Search items were defined for each risk factor based on the initial manual review.
4. Inclusion and exclusion criteria were established for each risk factor category based on clinical relevance, event definition (sudden cardiac death or ventricular arrhythmia), and study sample size.
5. All search terms and inclusion/exclusion criteria were reviewed and approved by all co-authors prior to screening.
6. NM performed the initial literature search, sequentially screening titles, abstracts, and full texts, for studies that met the pre-defined inclusion and exclusion criteria.
7. Studies meeting criteria were retained for use in data extraction and synthesis. Note that not all studies meeting criteria were ultimately referenced.

| Clinical Syndrome | **Sudden Cardiac Death in HFrEF** |
| --- | --- |
| Search terms (AND, OR, NOT) and truncation (wildcard characters like *) | (“Heart Failure”[Mesh] OR “heart failure” OR “HFrEF” OR “left ventricular dysfunction”) AND (“Sudden Cardiac Death”[Mesh] OR “sudden cardiac death” OR “sudden death” OR “arrhythmic death”) |
| Databases searched | *Medline* |
| Time period of search | *1/1/1975 – 8/1/2025* |
| Language | *English or translated into English* |
| Types of studies to be included | *Randomized controlled trials, cohort studies, case-control studies, and systematic reviews* |
| Inclusion criteria | *Population: Patients with HFrEF*  *Outcome: SCD and variables associated with SCD* |
| Exclusion criteria | Non-peer reviewed studies |
| Total results | 9,977 |
| Results meeting inclusion and exclusion criteria after title, abstract, and full text review | 215 |
| Selected high quality studies highlighted in Table 1 | 24 |

| **Risk Factor** | **Year** | **N (total patients)** | **% SCD with RF** | **% SCD without RF** | | **% with ICD** | |  |
| --- | --- | --- | --- | --- | --- | --- | --- | --- |
| **Syncope^1^** | 1993 | 491 | 45 | | 12 | | ≈0 (1 patient) | |
| **Abnormal CPET (VE/VCO2)^2^** | 2007 | 156 | 19 | | 6.8 | | 7.7 | |
| **Abnormal CPET (EOV)^2^** | 2007 | 156 | 33 | | 0 | | 7.7 | |
| **Etiology of HF^3^** | 2021 | 4,803 | 3 | | 1 | | 100 | |
| **LVEF^4^** | 2019 | 203,135 | 32 | | 18-20 | | est. <5.0 | |
| **Ventricular Arrhythmias (Previous VT/VF episode)^5^** | 2024 | 2,177 | 16 | | 8.1 | | 100 | |
| **Ventricular Arrhythmias (Non-sustained VT)^6^** | 2020 | 1,866 | 10-14 | | 4-5 | | 50 | |
| **Ventricular Arrhythmias (Inducibility on Electrophysiology Study)^7^** | 2000 | 2,202 | 32 | | 24 | | 5.6 | |
| **NT-proBNP^8^** | 2002 | 452 | 19 | | 1 | | 1.0 | |
| **Norepinephrine Levels^9^** | 2005 | 83 | 4.8 | | 1.3 | | 0 | |
| **Potassium Levels^10^** | 2022 | 3,398 | 24 | | 16 | | 4.0 | |
| **eGFR^11^** | 2024 | 1,676 | 23 | | 21 | | 2.8 | |
| **LMNA or Desmosomal Gene Mutation^12^** | 2019 | 487 | 43 | | 17 | | 40 | |
| **Heart Rate Turbulence^13^** | 2008 | 607 | 16 | | 6 | | 2.0 | |
| **ERP^14^** | 2013 | 132 | 38 | | 6 | | 2.3 | |
| **LBBB^15^** | 2002 | 5,517 | 8.3 | | 4.6 | | 0 | |
| **Multiple ECG Abnormalities^16^** | 2024 | 356 | 83 | | 17 | | 0 | |
| **ECG Abnormalities in NICM^17^** | 2011 | 572 | 47 | | 7.6 | | 1.9 | |
| **ECG Abnormalities in ICM^17^** | 2011 | 998 | 52 | | 6.4 | | 1.5 | |
| **LGE^18^** | 2018 | 7,882 | 10 | | 3.0 | | 13 | |
| **LGE + QRS duration^19^** | 2018 | 531 | 5.1 | | 1.2 | | 18 | |
| **LVEDD^20^** | 2014 | 747 | 83 | | 54 | | 6.0 | |
| **GLS^21^** | 2019 | 939 | 18 | | 2.0 | | 0 | |
| **Mechanical Dispersion^21^** | 2019 | 939 | 11 | | 5.8 | | 0 | |
| **ADMIRE-HF Score^22^** | 2020 | 90 | 50 | | 21 | | 21 | |
| **PARADIGM-HF Model^23^** | 2021 | 8,399 | 9.8 | | 3.6 | | 15 | |
| **ATMOSPHERE Model^24^** | 2021 | 7,156 | 9.7 | | 2.8 | | 0 | |
|  |  |  |  | |  | |  | |

**Table 2. Summary of Supplementary Information From Studies Evaluating SCD in HFrEF**
*Abbreviations: CPET, Cardiopulmonary Exercise Testing; ECG, Electrocardiogram; eGFR, Estimated Glomerular Filtration Rate; EOV, Exercise Oscillatory Ventilation; ERP, Early Repolarization Pattern; GLS, Global Longitudinal Strain; HF, Heart Failure; ICD, Implantable Cardioverter-Defibrillator; ICM, Ischemic Cardiomyopathy; LBBB, Left Bundle Branch Block; LGE, Late Gadolinium Enhancement; LVEF, Left Ventricular Ejection Fraction; LVEDD, Left Ventricular End-Diastolic Diameter; NICM, Non-Ischemic Cardiomyopathy; RF, Risk Factor; SCD, Sudden Cardiac Death; VCO2, Carbon Dioxide Output; VE, Minute Ventilation; VF, Ventricular Fibrillation; VT, Ventricular Tachycardia*

**References**

1. Middlekauff HR, Stevenson WG, Stevenson LW, Saxon LA. Syncope in advanced heart failure: high risk of sudden death regardless of origin of syncope. *J Am Coll Cardiol*. Jan 1993;21(1):110-6. doi:10.1016/0735-1097(93)90724-f

2. Guazzi M, Raimondo R, Vicenzi M, et al. Exercise oscillatory ventilation may predict sudden cardiac death in heart failure patients. *J Am Coll Cardiol*. Jul 24 2007;50(4):299-308. doi:10.1016/j.jacc.2007.03.042

3. Narins CR, Aktas MK, Chen AY, et al. Arrhythmic and Mortality Outcomes Among Ischemic Versus Nonischemic Cardiomyopathy Patients Receiving Primary ICD Therapy. *JACC: Clinical Electrophysiology*. 2022;8(1):1-11. doi:doi:10.1016/j.jacep.2021.06.020

4. Wehner GJ, Jing L, Haggerty CM, et al. Routinely reported ejection fraction and mortality in clinical practice: where does the nadir of risk lie? *Eur Heart J*. Mar 21 2020;41(12):1249-1257. doi:10.1093/eurheartj/ehz550

5. Rav-Acha M, Wube O, Brodie OT, et al. Evaluation of MADIT-II Risk Stratification Score Among Nationwide Registry of Heart Failure Patients With Primary Prevention Implantable Cardiac Defibrillators or Resynchronization Therapy Devices. *Am J Cardiol*. Jan 15 2024;211:17-28. doi:10.1016/j.amjcard.2023.10.044

6. Chatterjee NA, Rea TD. Secondary prevention of sudden cardiac death. *Heart Rhythm O2*. Oct 2020;1(4):297-310. doi:10.1016/j.hroo.2020.08.002

7. Buxton AE, Lee KL, DiCarlo L, et al. Electrophysiologic Testing to Identify Patients with Coronary Artery Disease Who Are at Risk for Sudden Death. *New England Journal of Medicine*. 2000;342(26):1937-1945. doi:doi:10.1056/NEJM200006293422602

8. Berger R, Huelsman M, Strecker K, et al. B-Type Natriuretic Peptide Predicts Sudden Death in Patients With Chronic Heart Failure. *Circulation*. 2002;105(20):2392-2397. doi:doi:10.1161/01.CIR.0000016642.15031.34

9. Peng YX, Shan J, Zhang SJ, et al. Cardiac risk stratification in patients with congestive heart failure: a catecholamines-beta-adrenoceptor-cAMP pathway. *Chin Med Sci J*. Jun 2005;20(2):93-8.

10. Miura Y, Higuchi S, Kohno T, et al. Association of Potassium Level at Discharge with Long-Term Mortality in Hospitalized Patients with Heart Failure. *J Clin Med*. Dec 11 2022;11(24)doi:10.3390/jcm11247358

11. Sobue Y, Watanabe E, Funato Y, Yanase M, Izawa H. Renal dysfunction is a time-varying risk predictor of sudden cardiac death in heart failure. *ESC Heart Failure*. 2024;11(5):3085-3094. doi:<https://doi.org/10.1002/ehf2.14892>

12. Gigli M, Merlo M, Graw SL, et al. Genetic Risk of Arrhythmic Phenotypes in Patients With Dilated Cardiomyopathy. *J Am Coll Cardiol*. Sep 17 2019;74(11):1480-1490. doi:10.1016/j.jacc.2019.06.072

13. Cygankiewicz I, Zareba W, Vazquez R, et al. Heart rate turbulence predicts all-cause mortality and sudden death in congestive heart failure patients. *Heart Rhythm*. 2008/08/01/ 2008;5(8):1095-1102. doi:<https://doi.org/10.1016/j.hrthm.2008.04.017>

14. FURUKAWA Y, YAMADA T, MORITA T, et al. Early Repolarization Pattern Associated with Sudden Cardiac Death: Long-Term Follow-Up in Patients with Chronic Heart Failure. *Journal of Cardiovascular Electrophysiology*. 2013;24(6):632-639. doi:<https://doi.org/10.1111/jce.12093>

15. Baldasseroni S, Opasich C, Gorini M, et al. Left bundle-branch block is associated with increased 1-year sudden and total mortality rate in 5517 outpatients with congestive heart failure: a report from the Italian network on congestive heart failure. *Am Heart J*. Mar 2002;143(3):398-405. doi:10.1067/mhj.2002.121264

16. Mashood F. ECG Risk Score Model to Predict SCD in HFrEF: Retrospective Review in a Tertiary Centre. *IIUM Medical Journal Malaysia*. 2024;23 No 4(October 2024)

17. Pei J, Li N, Gao Y, et al. The J wave and fragmented QRS complexes in inferior leads associated with sudden cardiac death in patients with chronic heart failure. *EP Europace*. 2012;14(8):1180-1187. doi:10.1093/europace/eur437

18. Ganesan AN, Gunton J, Nucifora G, McGavigan AD, Selvanayagam JB. Impact of Late Gadolinium Enhancement on mortality, sudden death and major adverse cardiovascular events in ischemic and nonischemic cardiomyopathy: A systematic review and meta-analysis. *International Journal of Cardiology*. 2018/03/01/ 2018;254:230-237. doi:<https://doi.org/10.1016/j.ijcard.2017.10.094>

19. Marume K, Noguchi T, Tateishi E, et al. Mortality and Sudden Cardiac Death Risk Stratification Using the Noninvasive Combination of Wide QRS Duration and Late Gadolinium Enhancement in Idiopathic Dilated Cardiomyopathy. *Circulation: Arrhythmia and Electrophysiology*. 2018/04/01 2018;11(4):e006233. doi:10.1161/CIRCEP.117.006233

20. Narayanan K, Reinier K, Teodorescu C, et al. Left ventricular diameter and risk stratification for sudden cardiac death. *J Am Heart Assoc*. Sep 16 2014;3(5):e001193. doi:10.1161/jaha.114.001193

21. Perry R, Patil S, Marx C, et al. Advanced Echocardiographic Imaging for Prediction of SCD in Moderate and Severe LV Systolic Function. *JACC Cardiovasc Imaging*. Feb 2020;13(2 Pt 2):604-612. doi:10.1016/j.jcmg.2019.07.026

22. Ikeda-Yorifuji I, Yamada T, Tamaki S, et al. Prediction of sudden cardiac death in chronic heart failure patients with reduced ejection fraction by ADMIRE-HF risk score and early repolarization pattern. *J Nucl Cardiol*. Jun 2020;27(3):992-1001. doi:10.1007/s12350-019-01639-6

23. Rohde LE, Vaduganathan M, Claggett BL, et al. Dynamic changes in cardiovascular and systemic parameters prior to sudden cardiac death in heart failure with reduced ejection fraction: a PARADIGM-HF analysis. *European Journal of Heart Failure*. 2021;23(8):1346-1356. doi:<https://doi.org/10.1002/ejhf.2120>

24. Shen L, Claggett BL, Jhund PS, et al. Development and external validation of prognostic models to predict sudden and pump-failure death in patients with HFrEF from PARADIGM-HF and ATMOSPHERE. *Clin Res Cardiol*. Aug 2021;110(8):1334-1349. doi:10.1007/s00392-021-01888-x
